# Cost-effectiveness analysis of atezolizumab in patients with non-small-cell lung cancer ineligible for treatment with a platinum-containing regimen: a United Kingdom health care perspective

**DOI:** 10.3389/fpubh.2023.1282374

**Published:** 2023-09-29

**Authors:** Yunlin Jiang, Mingye Zhao, Jiayi Xi, Jiaqi Li, Wenxi Tang, Xueping Zheng

**Affiliations:** ^1^Nanjing Hospital of Chinese Medicine Affiliated to Nanjing University of Chinese Medicine, Nanjing, China; ^2^Nanjing University of Chinese Medicine, Nanjing, China; ^3^Department of Pharmacoeconomics, School of International Pharmaceutical Business, China Pharmaceutical University, Nanjing, Jiangsu, China

**Keywords:** atezolizumab, non-small-cell lung cancer, UK, platinum-ineligible, cost-effectiveness, price simulation

## Abstract

**Background:**

Cost-effectiveness of atezolizumab, as a treatment for advanced non-small-cell lung cancer (NSCLC) patients who cannot receive a platinum-containing regimen,was still unknown. Our objective was to evaluate the cost-effectiveness of atezolizumab vs. chemotherapy in this indication from the perspective of UK healthcare system.

**Methods:**

From the global, randomised, open-label, phase III IPSOS trial, clinical inputs and patient characteristics were obtained. A partitioned survival model with three health states was built: Progression-free survival, progressed disease and death. A lifetime time horizon was applied, with an annual discount rate of 3.5%. Additionally, the willingness-to-pay threshold of £50,000/QALY was utilized. Primary outcomes were quality-adjusted life-year (QALY), costs, and incremental cost-effectiveness ratio (ICER). Sensitivity, scenario, and subgroup analyses were used to assess the reliability of base-case results. Price simulations were carried out in order to provide information for the pricing strategy at specific willingness-to-pay threshold.

**Results:**

In the base-case analysis, atezolizumab resulted in a gain of 0.28 QALYs and an ICER of £94,873/QALY compared to chemotherapy, demonstrating no cost-effectiveness. Price simulation results revealed that atezolizumab would be preferred at a price lower than £2,215 (a reduction of 41.8%) at the willingness-to-pay threshold of £50,000. Sensitivity, scenario and subgroup analyses revealed these conclusions were generally robust, the model was most sensitive to the price of atezolizumab and subsequent medication. Furthermore, atezolizumab was found to be more cost-effective for patients displaying a positive PD-L1 expression, with an ICER of £72,098/QALY as compared to chemotherapy.

**Conclusion:**

Atezolizumab is not cost-effective for patients with advanced NSCLC ineligible for platinum-containing regimen, potential price reduction is necessary.

## 1. Introduction

Globally, lung cancer is the second most frequently diagnosed cancer and is responsible for the most of cancer-related deaths. In the UK, lung cancer accounts for 13% of newly diagnosed cancer cases and is associated with 21% of cancer-related deaths (1, 2). With the process of aging, the prevalence of lung cancer has been on the rise. Non-small-cell lung cancer (NSCLC) accounts for the largest proportion among all types of lung cancer, with a staggering 88% prevalence. Additionally, over half of the patients are already in advanced stages at the time of diagnosis (3). Consequently, there is a substantial burden associated with lung cancer. In 2010, the overall expenses of lung cancer over a span of 5 years amounted to around £267 million in the UK. When considering value-based oncology, it becomes crucial to assess the relative cost-effectiveness of various treatment options (4).

For NSCLC patients with negative driver genes, the current standard treatment is platinum-based doublet chemotherapy, combined with immunotherapy and/or anti-angiogenic therapy (5). However, in the real clinical setting, a significant portion of patients cannot tolerate platinum-based chemotherapy. Initially, the majority of NSCLC patients in the real world are diagnosed with an Eastern Cooperative Oncology Group (ECOG) performance status (PS) score of ≥2. In the UK, it is estimated that 53% of lung cancer patients have an ECOG PS score of ≥2 (6). In most of clinical studies focused on immunotherapy, ECOG PS-high-score and older adult patients were excluded (7, 8). Regardless of the type of treatment received, patients with an ECOG PS score ≥ 2 had a worse prognosis compared to patients with a PS score of 0–1 (9–11). Secondly, statistics showed that the average age of onset for NSCLC patients is >70 years old (1). Overall, these patients usually have some comorbidities or contraindications that make them unsuitable for platinum-based chemotherapy. For NSCLC patients who cannot tolerate platinum-based chemotherapy, recommended treatments included combination therapy, monotherapy, or palliative care (12). With such treatments, the median survival time for patients was only 9.2–9.5 months (13, 14). Therefore, it is necessary to investigate strategies that offer enhanced effectiveness and safety for individuals within these patients.

The IPSOS trial is the first and only global phase III randomized controlled validation study conducted in a population not suitable for receiving platinum-based doublet chemotherapy (14). In 23 countries across Europe, Asia, and America, the research was carried out in 91 regions. Patients who met the criteria were randomly divided into two groups, with a ratio of 2:1. One group received atezolizumab (*n* = 302), while the other group received chemotherapy (*n* = 151). The objective of this study was to assess the efficacy and safety of atezolizumab as the initial treatment among these patients (more details are provided in Supplementary Table S1). The results showed that atezolizumab significantly reduced the death risk by 22% and also decreased the risk of disease progression by 13%, which suggested that atezolizumab was a potential first-line choice for advanced NSCLC patients who cannot undergo platinum-based chemotherapy.

Atezolizumab has been approved in the UK for the treatment of five indications of NSCLC (15). (1) Adjuvant treatment for patients with stage II to IIIA NSCLC who have a high PD-L1 expression level. (2) It is recommended to be added to bevacizumab, paclitaxel, and carboplatin or nab-paclitaxel and carboplatin, as the first-line treatment for patients with advanced non-squamous NSCLC. (3) The initial therapy for adult patients with metastatic NSCLC having PD-L1 expression of 10% or more is recommended. (4) Second-line treatment for patients diagnosed with locally advanced or metastatic NSCLC, who have undergone chemotherapy previously. The disease burden for the patients of interest is significant (6), considering the IPSOS trial results were published in July 2023 in the Lancet, it is expected that the indication of atelizumab for platinum-based chemotherapy intolerant population would be expedited for approval in the UK (14). In order to offer new, effective, and safe treatment options for advanced NSCLC patients who are ineligible for treatment with a platinum-containing regimen, as well as provide a more economical solution to alleviate their disease-related financial burden and ensure the optimal allocation of limited health resources, clinicians and decision-makers need information on cost-effectiveness to make informed healthcare decisions. Therefore, we aimed to inform decision-makers about the cost-effectiveness of atezolizumab in the UK healthcare system. The analysis was conducted from the UK healthcare system perspective to provide evidence for health technology assessment submissions and establish drug pricing strategies.

## 2. Materials and methods

### 2.1. Model overview

The purpose of this cost-effectiveness analysis was to compare the clinical and economic outcomes of atezolizumab with chemotherapy in patients with advanced NSCLC who cannot receive a platinum-containing regimen.

A partitioned survival model was created, comprising three health states: progression-free survival (PFS), progressed disease (PD), and death (Supplementary Figure S1). A 10-year lifetime horizon was applied. The percentage of patients who were alive or free from progression at each cycle (cycle length: 21 days) were estimated using the areas under the OS or PFS curves. By calculating the difference between the OS and PFS curves, the PD rate was determined. In the PFS state, patients were administered treatment using either atezolizumab or single-agent chemotherapy (vinorelbine or gemcitabine). Once the disease progressed or drug-discontinuation, patients would receive subsequent treatments consistent with the IPSOS trial (14). For patients receiving no subsequent anti-cancer treatments, best supportive care (BSC) was performed, more details are available in the Supplementary Table S2.

According to the National Institute for Health and Care Excellence (NICE) reference case, the model was developed from the aspect of the UK National Health Service (NHS) and personal social services (16). Both costs and utilities were discounted annually at a rate of 3.5% (17). The threshold for willingness-to-pay was established at £50,000 for each quality-adjusted life-year (QALY), taking into consideration the population of interest receiving “end-of-life” treatment, as recommended by the NICE (18). Additional analyses were conducted using thresholds of £30,000 per QALY and £90,000 per QALY. The reported outcomes included costs, life-years, QALY, and incremental cost-effectiveness ratio (ICER).

### 2.2. Population and health state transitions

Clinical inputs and patient characteristics were all extracted from the IPSOS trial (14). The population of interest were individuals with stage IIIB or stage IV NSCLC, who were ineligible for platinum-based therapy, had an ECOG PS of 2–3, mean age of 75 years, and possessed wild-type EGFR or ALK gene mutations. Besides, males comprised 73% of the population. In addition, according to a NICE technology appraisal (17), the parameters assumed for the population in this model were a mean weight of 74.1 kg, an average height of 170 cm, and a body surface area of 1.85 m^2^.

The OS and PFS probabilities were derived from the Kaplan–Meier curves documented in the IPSOS trial (14, 19). The required data was extracted using the GetData Graph Digitizer software. Guyot’s method was utilized to reconstruct the estimates of individual patient data (IPD) (20). The accuracy of the IPD reconstruction was assessed using the root mean square error (RMSE). The RMSEs of reconstructed IPDs were ranging from 0.002 to 0.006, which indicated a high accuracy. Subsequently, the reconstructed IPD was utilized to fit various parametric functions, including exponential, gamma, Gompertz, Weibull, generalized gamma, log-normal, log-logistic, fractional polynomial (FP), restricted cubic spline (RCS), and Royston-Parmar spline (RP) models. The goodness-of-fit criteria for model selection were evaluated based on the Akaike information criterion (AIC) and visual inspection (21).

Furthermore, age-based adjustments were made to the mortality rate to ensure it would not fall below that of the general population in the UK (22).

We opted for the Gompertz model for OS and RP-hazard models for PFS of atezolizumab. As for the PFS and OS of chemotherapy, we selected the RP-odds and Log-normal models. Detailed parameters are presented in Supplementary Table S3. Further information of goodness-of-ft results can be found in Supplementary Table S4 and Supplementary Figure S2.

### 2.3. Adverse events

Adverse events (AEs) rates were extracted from the IPSOS trial (14), considering only AEs of grade 3 or higher with an incidence exceeding 1% in either group. These events encompassed dyspnoea, anemia, neutropenia, leukopenia, nausea, vomiting, rash, decreased white blood cell count, and decreased neutrophil count. For additional details regarding incidences, durations and costs, refer to Supplementary Tables S5, S7.

### 2.4. Treatment duration

As per the clinical trial design, we assumed that treatment would continue until disease progression or unacceptable toxicity occurred. Median treatment duration for patients receiving atezolizumab and chemotherapy were 6 and 4 cycles, respectively. Observations revealed discontinuation rates of 13% for atezolizumab and 14% for chemotherapy (14). Since specific discontinuation times were not available for each patient, we employed the DEALE method to estimate the cyclical rate (23). The cyclical discontinuation rates for atezolizumab and chemotherapy were 2.3 and 3.7%, respectively.

### 2.5. Health state utilities

A disutility approach was used, which took into account the decrease in utility as age and gender increase, based on the population norms of the UK EQ-5D-3L (24). The decrement can be summarized as follows:


$$\mathrm{Utility}=0.951+0.0212\times \mathrm{Male}-0.000259\times \mathrm{Age}-0.000032\times {\mathrm{Age}}^{2}$$

The reported utility values in each study were used to calculate the disutility associated with each health state by subtracting it from the general population utility. Then, these disutility values were deducted from the population norms. The base-case analysis utilized the disutilities reported from IMpover150 (25), a study that examined the treatment of metastatic non-squamous NSCLC with atezolizumab. Using EQ-5D questionnaire, The utility values for PFS and PD when undergoing treatment were calculated as 0.71 and 0.69, respectively. The disutilities of PFS and PD when not receiving treatment were obtained from van den Hout’s research (26). Likewise, it was observed that the patients in van den Hout’s study adequately represented the population of interest. Disutilities of AEs were included, and values were all from NICE committee papers, the duration-adjusted negative effects caused by AEs were assumed to occur during the initial cycle (27–29). More details can be found in Supplementary Tables S6, S7.

### 2.6. Treatment unit costs and health state costs

Only direct medical costs were considered in our study, encompassed the costs of acquiring active-treatment drugs and follow-up items, along with the expenses of AE management, BSC, and end-of-life care. The prices of generic drugs were obtained from the electronic market information tool (eMIT) for the year 2022 (30). Prices of medications were obtained from the listed price outlined in the 2022 British National Formulary (BNF) (31). We assumed complete vial sharing for all weight-based medications, as a conservative estimate (4). In this study, we utilized the NHS 2021–2022 reference cost (code SB12Z) (32) to calculate administration costs (£287/cycle) for all intravenous drugs (See more in Supplementary Tables S8, S9). Due to the lack of reported by the IPSOS trial regarding the usages of vinorelbine and gemcitabine, we assumed that in our base-case analysis, 50% of the patients received intravenous vinorelbine, while the remaining patients received gemcitabine. Besides, dosing intensity was assumed to be 100% for both groups due to lack of report. Healthcare resource utilized during the follow-up period included CT chest scan, chest radiography, electrocardiogram, outpatient visit, community nurse, clinical nurse specialist, general practitioner surgery, general practitioner home visit, and therapist visit. Prices of follow-up items were obtained from 2021–2022 NHS reference costs and the 2022 Personal Social Services Research Unit (PSSRU) costs (33). Follow-up care costs were £326 and £506 per cycle for PFS and PD in this model, respectively, more information is available in Supplementary Table S10. AE management costs were obtained from NHS or NICE committee papers targeting on advanced NSCLC (Supplementary Table S5) (17, 34). BSC was consisted by radiotherapy, morphine, bisphosphonate, steroids, nonsteroidal anti-inflammatory drugs, denosumab and dietitian, doses and prices of above items were taken from NHS, BNF or eMIT. In our base-case analysis, cost for BSC was £379 per cycle. The costs of end-of-life care was considered a one-time expense. Based on a NICE committee paper (17), the average cost per episode of end-of-life care in our model was £4,773, details for costs of BSC and end-of-life care are available in Supplementary Table S11. The prices of all mentioned items were adjusted to 2022 using the PSSRU annual inflation hospital and community health services index.

### 2.7. Sensitivity analyses

We conducted deterministic sensitivity analysis (DSA) to examine the impact of crucial parameters on the ICER, and the findings were presented as tornado diagrams. All parameters were modified either within the designated 95% confidence intervals (CI) or by ranging the base-case values (±20%). Detailed sources of uncertainty are provided in Supplementary Table 12.

A Monte Carlo simulation was performed with 10,000 iterations to conduct probabilistic sensitivity analysis (PSA) on the base-case. Additionally, we conducted 1,000 iterations for the PSA of scenario and subgroup analyses. For cost, we opted for the gamma distribution, while for probability, proportion, and utility, we chose the beta distribution. The scatter plots were utilized to visually present the outcomes of the base-case PSA. Afterwards, probability of being cost-effectiveness at the willingness-to-pay threshold ranged from £0 to £150,000 was tested by utilizing cost-effectiveness acceptability curves (CEAC).

### 2.8. Scenario analysis

In this study, we conducted scenario analyses considering uncertainties in model structure and parameters, such as uncertainty in survival data extrapolation, patient medication adherence, medication patterns, and in medication duration, and heterogeneity of utility values.

1. In scenario 1, we only considered standard parametric survival models.
2. In scenario 2, the utility values of PFS and PD states reported in the IMpover110 trial (PFS: 0.76; PD, 0.69) were utilized.
3. In scenario 3, dosing intensity for both atezolizumab and chemotherapy was assumed to be the same as the IMpover110 trial (35).
4. In scenario 4, it is assumed that patients take vinorelbine orally.
5. In scenario 5, active treatment during the PD state persisted until 3 months prior to death.
6. In scenario 6, we adjusted the utilization ratio of vinorelbine or gemcitabine within the range of 0–100%.

### 2.9. Subgroup analysis

The ICER, probability of being cost-effective at the selected willingness-to-pay threshold, and cost of being cost-effective at the chosen willingness-to-pay threshold for atezolizumab in each subgroup were calculated using subgroup-specific hazard ratios (HRs) of PFS and OS based on Cox proportional hazards models. We considered the subgroup factors of age (≥80, 70–79, or < 70), sex (male or female), race (white or Asian), ECOG PS score (0–1, 2, or 3), tobacco use history (previous, current, or never), histology (non-squamous or squamous), stage (IIIB or IV), brain or liver metastases (yes or no), number of metastatic sites (≥3 or < 3) and PD-L1 expression level (<1, 1–49%, or > 50%).

### 2.10. Price simulation

The price simulation analysis incorporated fluctuating prices ranging from £1,000 to £3,800, with increments of £10, as per the results from our base-case analysis. Furthermore, Monte Carlo simulation of 1,000 iterations were performed to conduct PSAs for each respective price.

The values, ranges, and sources for all parameters utilized in this model are summarized in Supplementary Table S12.

## 3. Results

### 3.1. Model validation

The model’s face validity, encompassing its structure, assumptions, data sources, and results, underwent evaluation by clinical experts. The validation results demonstrated a strong fit of our model, as the survival rates for both PFS and OS were consistent with the original data obtained from the IPSOS trial (Supplementary Figure S2). 4-year OS or PFS rates of both atezolizumab and chemotherapy were less than 10%, indicated little uncertainty regards extrapolation (14).

### 3.2. Base-case analysis results

To conclude, Atezolizumab is not an economical option for patients with advanced NSCLC ineligible for treatment with a platinum-containing regimen as compared to chemotherapy at the current price of £3807.69/1,200 mg. Atezolizumab can be deemed cost-effective only when priced below £2215/1,200 mg at the willingness-to-pay threshold of £50,000/QALY.

The findings of the base-case analysis are outlined in Table 1. The lifetime costs for atezolizumab and chemotherapy amounted to £56,950 and £30,744, respectively. Atezolizumab exhibited a gain of 0.46 life-years and 0.28 QALYs in contrast to chemotherapy. The ICER of atezolizumab compared to chemotherapy were £94,873/QALY, which was higher than the recommended willingness-to-pay threshold of £50,000/QALY, indicating that atezolizumab was not cost-effective when compared to chemotherapy at the current price of £3807.69/1,200 mg. We also conducted an alternative analysis focusing solely on PFS. The ICER for atezolizumab compared to chemotherapy was £213,196/QALY. Breakdown results of costs are provided in Supplementary Table S13.

**Table 1:** Results of base-case analysis.

Model	Drug	*Cum* cost (£)	*Cum* life years	*Cum* effect (QALY)	Incremental cost (£)	Incremental effect (QALY)	ICER (£/QALY)
OS	Chemotherapy	30,744	1.07	0.58			
	Atezolizumab	56,950	1.53	0.86	26,206	0.28	94,873
PFS	Chemotherapy	10,325	0.54	0.24			
	Atezolizumab	40,209	0.77	0.38	29,884	0.14	213,196

Cum, cumulative, ICER incremental cost-effectiveness ratio, OS overall survival, PFS progression-free survival, QALY quality-adjusted life year.

### 3.3. Sensitivity analyses

**Figure 1:**
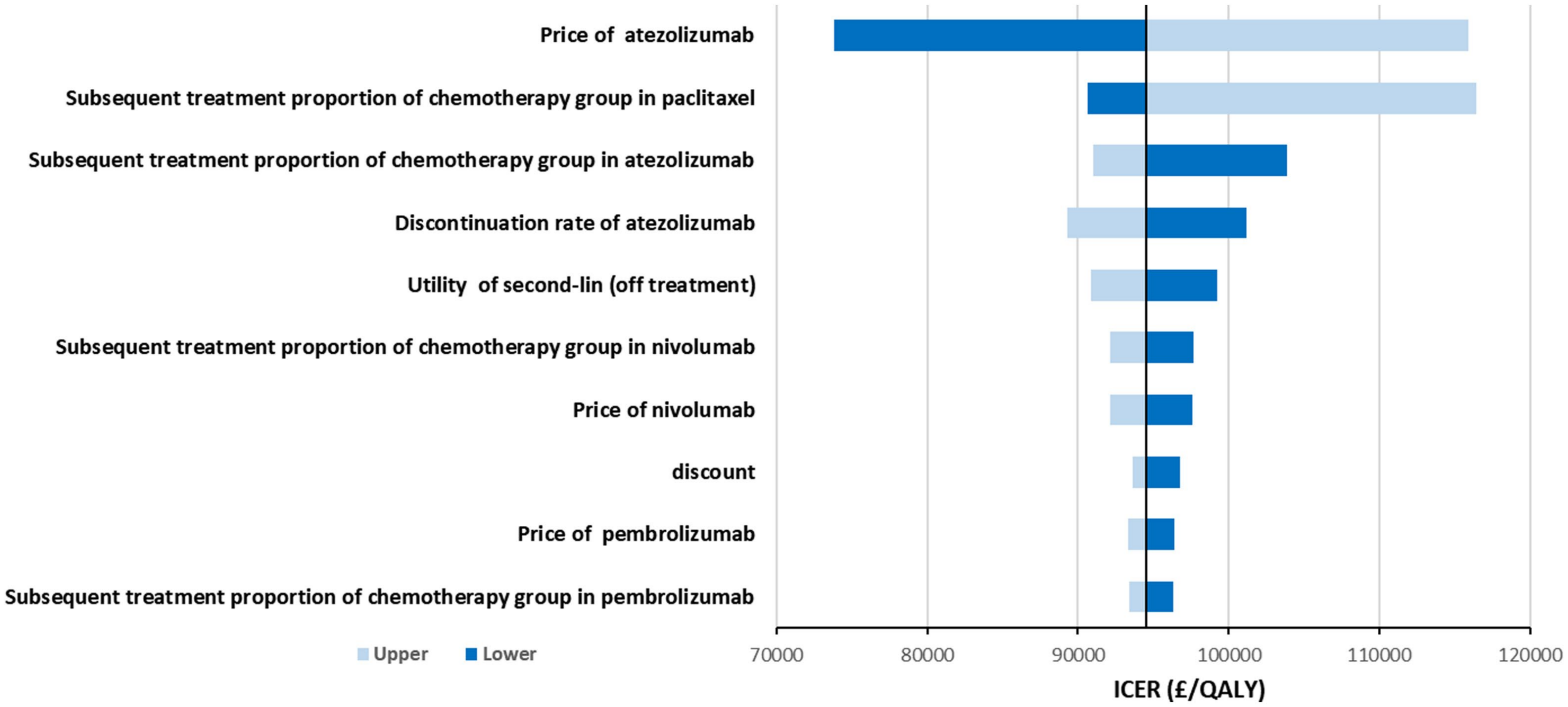
Tornado diagram shows the association of variables with the ICER of atezolizumab vs. chemotherapy.

The results of the DSA are shown in Figure 1. The variables that had the greatest impact on the ICER were the price of atezolizumab, percents of patients received subsequent paclitaxel and atezolizumab, discontinuation rate of atezolizumab, and the utility of PD. The price of atezolizumab had the greatest impact on ICER, but in this part, we only considered a limited range of fluctuations, which meant that even at the lowest price, atezolizumab was still not cost-effective. The proportion of patients receiving immune checkpoint inhibitors and other drugs after progression also had a significant impact on ICER, mainly due to the high price of these drugs. Similarly, discontinuation rates related to patient compliance and medication safety were significant factors affecting ICER. Additionally, the impact of utility value on ICER could not be ignored, as it was clearly related to the patient’s effectiveness. Lastly, the influence of discount rates was unquestionable, as they were closely related to the results of output and input indicators. Overall, after allowing parameter fluctuated within the specified upper and lower limits, it was established that atezolizumab was unlikely to exhibit cost-effectiveness at the threshold of £50,000/QALY. Nevertheless, this conclusion may be reevaluated if the threshold was set at £90,000/QALY.

**Figure 2:**
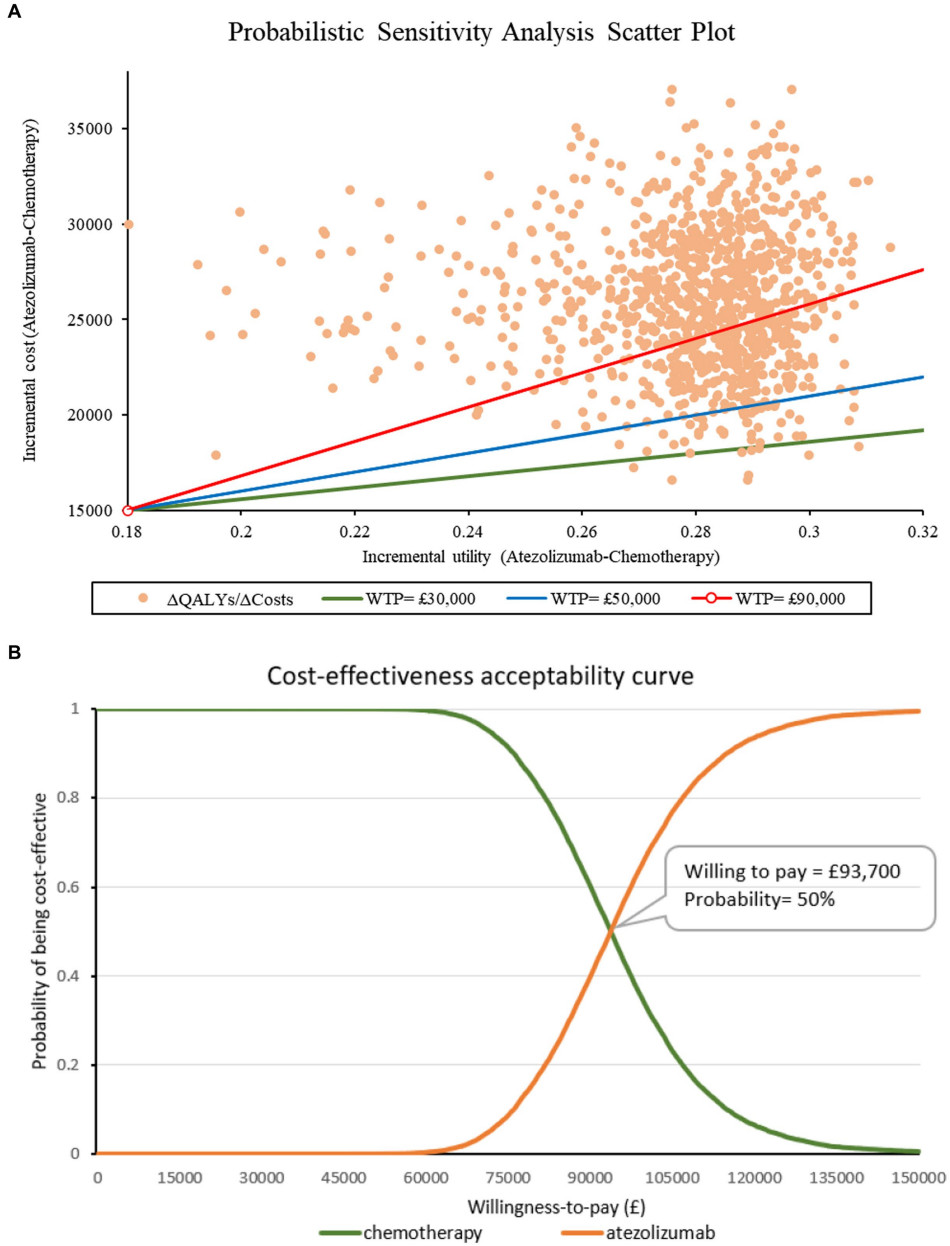
Results of the probabilistic sensitivity analysis.

The scatter plot and CEAC curve can be found in Figure 2. The results from the PSA showed that the average cost of atezolizumab was £56,943 and the average cost of chemotherapy was £30,754. Moreover, the average effects for these two drugs are 0.86 and 0.58 QALYs, respectively. Following 10,000 iterations, the average ICER is calculated to be £94,384. Atezolizumab was considered to be cost-ineffective at the threshold of £50,000/QALY. Even with a higher threshold of £90,000/QALY, the probability of atezolizumab being cost-effective remained at 40%. The CEAC curve suggested that atezolizumab would be cost-effective if the threshold surpasses £93,700/QALY. Nevertheless, attaining this threshold within the present healthcare landscape in the UK poses significant challenges.

Sensitivity analyses results validated the base-case conclusion. At the threshold of £50,000/QALY, atezolizumab was not cost-effective at the current public price.

### 3.4. Scenario analysis

In general, the uncertainty related to the structural assumptions and parameter estimates examined had a negligible impact on the base-case conclusion. In scenario 1, utilizing the approaches commonly used that solely incorporate standard parametric models, the findings revealed that the ICER of atezolizumab in comparison to chemotherapy amounted to £99,040/QALY; In scenario 2, employing the utility values documented by the IMpover110 trial, the ICER was £90,974/QALY; In scenario 3, by modifying the dosing intensity for both drugs, the ICER amounted to £88,219/QALY; The ICER of atezolizumab against chemotherapy was £97,354/QALY in scenario 4, where the duration of second-line treatment was altered; In scenario 5, vinorelbine was administered orally, the ICER was £93,335/QALY; In scenario 6, when the usage ratio of gemcitabine ranged from 0 to 100%, the ICER ranged from £92,000 to £98,000/QALY. All scenarios resulted in comparable ICERs and reached the same conclusion. More details, refer to Supplementary Table S14 and Supplementary Figure S3.

### 3.5. Subgroup analysis

The subgroup analysis results are summarized in Table 2. Overall, the ICERs showed a significant association with HRs, indicating improved outcomes with lower risks of disease progression and death. The ICER for atezolizumab compared to chemotherapy in the entire patient cohort was £120,124/QALY. To meet the cost-effectiveness threshold of £50,000/QALY, the price would need to be £1,970. Atezolizumab did not show favorable results in any of the subgroups at the £50,000/QALY threshold. Atezolizumab was found to be more cost-effective for patients with positive PD-L1 expression (ICER: £72,098/QALY). However, atezolizumab performed worse in patients with PD-L1 expression levels ranging from 1 to 49%, as well as in female patients and those with liver or brain metastases. For other factors, such as age, sex, race, ECOG PS, tobacco use history, histology, number of metastatic sites, and disease stage, did not affect the conclusion, and ICER for atezolizumab compared to chemotherapy in all of these subgroups were over £100,000/QALY. Further details are in Table 2.

**Table 2:** Summary results for subgroup analyses.

Subgroup	ICER (£/QALY)	Price of atezolizumab^ab^ (£) being CE at a WTP of 30,000	Price of atezolizumab^ab^ (£) being CE at a WTP of 50,000	Price of atezolizumab^ab^ (£) being CE at a WTP of 90,000	Probability of atezolizumab^ab^ being CE at a WTP of 30,000	Probability of atezolizumab^ab^ being CE at a WTP of 50,000	Probability of atezolizumab^ab^ being CE at a WTP of 90,000
All patients	120,124	1,440	1970	3,015	0	0.003	0.229
*Age*							
≥80	108,555	1,135	2,110	3,265	0.001	0.1	0.388
70–80	306,696	1,210	1,390	1725	0	0.001	0.071
<70	238,188	1,275	1,520	2010	0	0.02	0.177
*Sex*							
Male	123,542	1,270	1860	2,925	0	0.002	0.225
Female	D	NA	NA	NA	NA	NA	NA
*Race*							
White	311,108	1,360	1,530	1875	0	0	0.069
Asian	120,995	2,965	1875	2,875	0	0.057	0.349
*ECOG PS*							
0/1	125,307	1,250	1810	2,850	0.005	0.093	0.328
2	251,359	1,360	1,585	2025	0	0.003	0.074
3	397,937	1,245	1,380	1,660	0.021	0.131	0.283
*Stage*							
IIIB	98,055	1,335	2050	3,520	0.002	0.152	0.433
IV	205,944	1,340	1,610	2,175	0	0	0.074
*Tobacco use history*							
Previous	187,340	1,360	1,665	2,295	0	0.002	0.114
Current	110,272	1,280	1910	3,165	0	0.102	0.388
Never	367,872	1,360	1,320	1,215	0.006	0.071	0.222
*Histology*							
Non-squamous	215,465	1,290	1,565	2,115	0	0	0.119
Squamous	136,042	1,365	1820	2,745	0	0.015	0.248
*Brain metastases*							
Yes	D	NA	NA	NA	NA	NA	NA
No	163,656	1,325	1700	2,450	0.025	0.099	0.22
*Liver metastases*							
Yes	D	NA	NA	NA	NA	NA	NA
No	127,801	1,350	1850	2,860	0	0.005	0.218
*Number of metastatic sites*							
<3	126,383	1,325	1850	2,865	0	0.012	0.281
≥3	337,969	1,280	1,450	1770	0	0.004	0.096
*PD-L1 expression level*							
<1%	293,190	1,310	1,500	1885	0	0.011	0.13
1–49%	D	NA	NA	NA	NA	NA	NA
≥50%	72,098	1,565	2,625	4,770	0	0.23	0.611

a The unit is £/1,200 mg.

b with a probability of 50% to be cost-effective.

CE, cost-effective; D, dominated; WTP willingness to pay.

### 3.6. Price simulation

**Figure 3:**
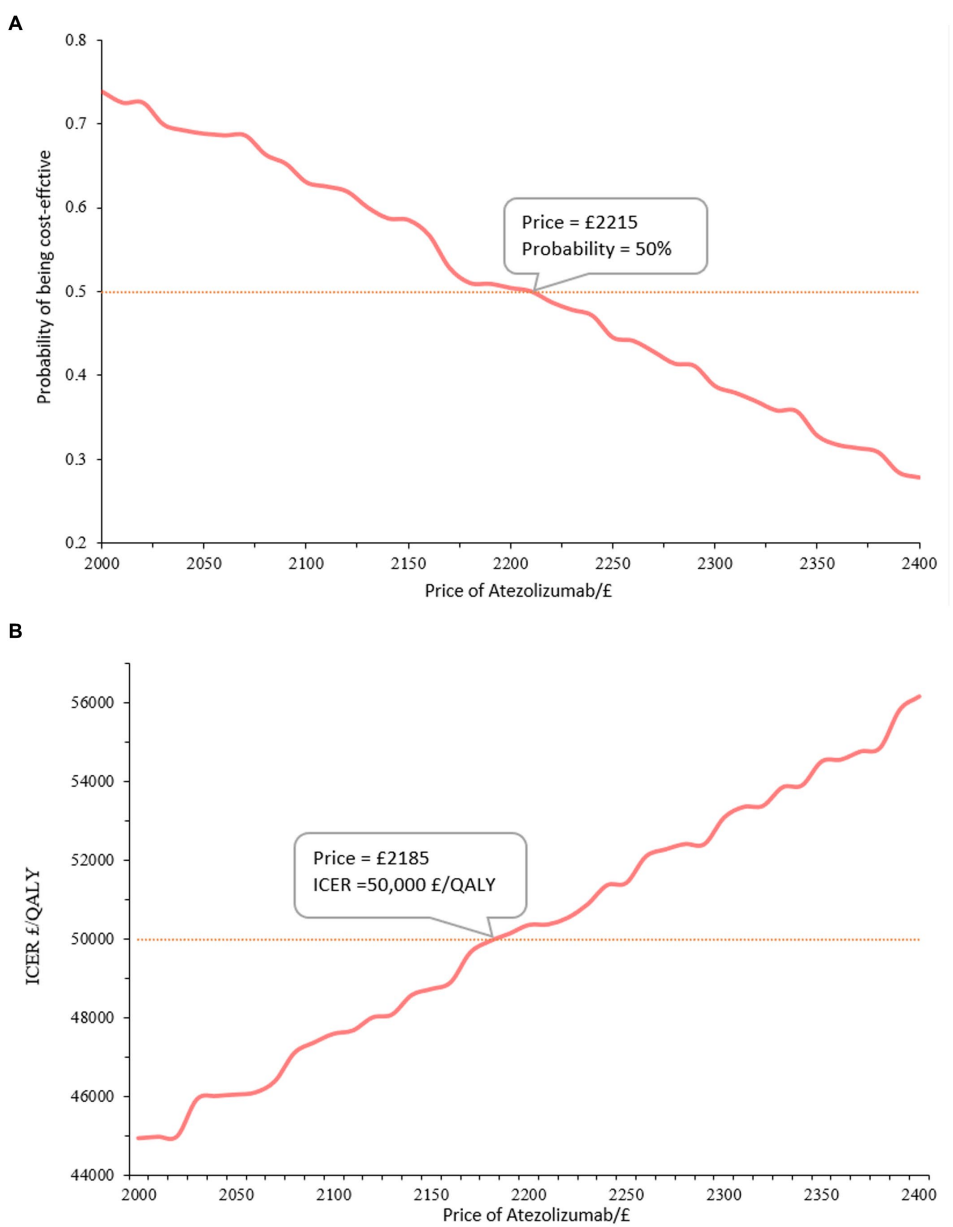
Main results of price simulation.

Overall, there is a positive correlation between the cost of atezolizumab and the ICER when compared to chemotherapy. It was concluded that atezolizumab was considered cost-effective if priced below £2,215/1,200 mg, given the willingness-to-pay threshold of £50,000/QALY. Atezolizumab was deemed cost-effective when the price was below £1,465 at a £30,000/QALY threshold. Additionally, for a price of £3,640, atezolizumab was considered cost-effective when the threshold was £90,000/QALY. More findings are depicted in Figure 3 and Table 3. There has been approved Patient Access Scheme for atezolizumab currently in the UK for several indications including advanced NSCLC (17), we believe that implementing the recommended price reductions for atezolizumab for NSCLC patients who are ineligible for treatment with a platinum-containing regimen would be practicality and feasible.

**Table 3:** Willingness-to-pay threshold of atezolizumab being cost-effective at specific price.

Price of atezolizumab^a^ (£)	WTP^b^ (£/QALY)	Price of atezolizumab^a^ (£)	WTP^b^ (£/QALY)	Price of atezolizumab^a^ (£)	WTP^b^ (£/QALY)
3,800	94,660	2,800	66,992	1800	39,324
3,700	91,893	2,700	64,225	1700	36,557
3,600	89,126	2,600	61,458	1,600	33,790
3,500	86,359	2,500	58,691	1,500	31,023
3,400	83,593	2,400	55,924	1,400	28,256
3,300	80,826	2,300	53,158	1,300	25,490
3,200	78,059	**2,200**	**50,391**	1,200	22,723
3,100	75,292	**2,100**	**47,624**	1,100	19,956
3,000	72,525	2000	44,857	1,000	17,189
2,900	69,759	1900	42,090		

a The unit is £/1,200 mg.

b With a probability of 50% to be cost-effective.

WTP willingness to pay.

## 4. Discussion

Atezolizumab was the first to demonstrate a significant enhancement in the survival of patients with advanced lung cancer who have an intolerance to platinum-based chemotherapy (14), irrespective of the type of pathology, PD-L1 expression, or PS score. The real-world NEJ057 study (13) also corroborated this finding. Immunotherapy regimens had significantly higher median OS compared to chemotherapy (19.8 months vs. 9.5 months). Regarding toxicity, findings from both the IPSOS and NEJ057 studies suggested that using immune-mono therapy could significantly decrease the occurrence of severe AEs. Based on the IPSOS trial, it was found that 16% of patients receiving atezolizumab experienced treatment-related severe AEs, while the figure for patients receiving chemotherapy is 33%. Clinical treatment guidelines and strategies is expected to enter a new era of immune-mono therapy. Considering the exorbitant price of atezolizumab compared to the standard treatments, physicians and patients confront the challenge of evaluating its cost-effectiveness. The escalating healthcare costs justify concerns regarding value-based oncology.

This study aimed to fulfill the unmet need for an economic assessment of this novel indication. According to our analysis, atezolizumab was found to be less favorable compared to chemotherapy. When considering a WTP threshold of £50,000/QALY over a lifetime time horizon, atezolizumab incurred an extra cost of £26,206 and had an additional effect of 0.28 QALYs. This led to an ICER of £94,873/QALY in comparison to chemotherapy. Considering parameters’ uncertainty, we used the 95% CI as their range of variation. For parameters with only standard deviation or errors, we assume to calculate a 95% CI based on their distribution. For parameters with standard deviation or confidence interval, we assume they vary within a range of ±20%. The findings from the sensitivity analyses provided evidence that the results of the base-case analysis were generally stable and reliable. The factors that had the greatest impacts on economic outcomes were the price of atezolizumab, second-line medication, discontinuation rate of atezolizumab, and utility for PD. To address the uncertainties around the structural assumptions and parameter estimates, multiple scenario analyses were conducted. The selection of survival models, dosing patterns, utility values, and other factors minimally influenced the base-case results and yielded consistent conclusions. Nevertheless, for patients with positive PD-L1 expression, atezolizumab performed better, with an ICER of £72,079/QALY compared to chemotherapy. While for patients with a PD-L1 expression level of 1–49%, female patients, and those with liver or brain metastases, atezolizumab could be cost-effective only when its unit cost lower than chemotherapy. The high diversity of subgroup results reminds us that patient characteristics during the administration of drugs in clinical practice is crucial for utilizing healthcare resources in a rational manner.

Based on the results presented above, price simulations were conducted to explore suitable pricing for atezolizumab for studied patients. It was found that atezolizumab would be cost-efective at a price of £2,215/1,200 mg (a reduction of 41.8%) at the threshold of £50,000/QALY. For patients with positive PD-L1 expression, atezolizumab would be cost-efective at a price of £2,625/1,200 mg (a reduction of 31.1%). At the threshold of £90,000/QALY, atezolizumab would be cost-effective after a 4.4% price reduction for overall patients, and atezolizumab was economical at the current price for PD-L1 positive patients. The benefit of atezolizumab in PD-L1 positive patients was also observed in those with stage II-IIIA NSCLC in the adjuvant setting (4) and those with metastatic NSCLC receiving first- or second-line treatment (36, 37). Nevertheless, atezolizumab was deemed less cost-effective for patients with liver or brain metastases.

The direct and indirect costs associated with advanced NSCLC, particularly as the disease progresses, place a significant financial burden on healthcare systems, society, patients, and caregivers. Therefore, it is of utmost importance to develop newer, more economical, and safer treatments for NSCLC that can effectively slow down or halt the progression of the disease. The well-known fact is that chemotherapy has limited practicality in advanced NSCLC patients, especially in those who are ineligible for treatment with a platinum-containing regimen. Our findings suggest that atezolizumab seems to be a potential choice. However, the current price is a deterrent to it becoming the standard of care (SOC). Therefore, price concession is necessary, and our research provide reference for decision-makers. Overall, this research contributes to the development of a new SOC for patients with NSCLC who are unable to tolerate the AEs of the most powerful treatments, and to expedite the approval of this SOC in the UK, thereby providing new treatment options for patients and helping to alleviate the economic burden associated with the disease. Through this study, we aim to inform the UK decision-makers that atezolizumab has the potential to be a new SOC for NSCLC patients who are ineligible for treatment with a platinum-containing regimen. However, at current pricing, it is not yet a cost-effective choice. Furthermore, atezolizumab can be deemed cost-effective only when priced below £2215/1,200 mg at the willingness-to-pay threshold of £50,000/QALY.

The model structure and approach employed in this study are in line with the NICE appraisal of atezolizumab monotherapy for untreated PD-L1 positive metastatic NSCLC (36). For accuracy, reliable sources of information such as NHS reference costs, the PSSRU, and eMIT were utilized. The strengths of this study lie primarily in the innovative research topic, high-quality clinical data, the consideration of various scenarios, and the extensive sensitivity analysis. As far as we know, no other analysis has evaluated the cost-effectivenes of atezolizumab for patients with advanced NSCLC who cannot receive a platinum-containing regimen. Additionally, we performed price simulations to offer decision-makers a more comprehensive comprehension of the economic value attached to atezolizumab. Further discussion is necessary due to the implications of this study. Decision-makers in the UK should be informed about the price at which atezolizumab would be deemed cost-effective for patients with advanced NSCLC who cannot receive treatment containing platinum. Furthermore, our evidence may support the UK health technology assessment submissions for this indication. Finally, our analysis explored the cost-effectiveness outcomes of the 28 prespecified subgroups in the IPSOS trial. NSCLC is in the era of precision treatment (38), economic information for the subgroups may assist in tailoring treatment choices.

Our study has several limitations. First, lack of individual data compelling us to make assumptions about proportional hazards in subgroup analyses. This may cause bias in the calculation of survival rate, thereby leading to errors in the results of subgroup analyses; Second, omitting grade 1 or 2 AEs may have introduced biases, causing the actual cost of treatments to be underestimated. Nevertheless, the sensitivity analysis results indicated that this limitation had minimal impact. Third, at this stage, we did not study the availability and affordability, implying that further research is required. Fourth, as the lack of report in the IPSOS trial (14), the incidences of all grade 3–5 AEs were unavailable. Instead, we could solely consider AEs of any grade with an incidence difference exceeding 5% between groups. This theoretically might result in the underestimation of costs. We contend that its impact was restricted given that the grade 3–5 AEs integrated into our model closely resembled those in the IPSOS trial (14). Our model included 83 events, whereas the IPSOS trial documented 84 events. Fifth, EQ-5D based utility was not collected in the IPSOS trial, and the varying information used for the utility values from different trials might have an influence on the outcomes. Despite conducting a scenario analysis, the impact is still uncertain.

## 5. Conclusion

From the perspective of the UK healthcare system, atezolizumab is not an economical option for patients with advanced NSCLC ineligible for treatment with a platinum-containing regimen. Moreover, atezolizumab can be deemed cost-effective only when priced below £2215/1,200 mg at the willingness-to-pay threshold of £50,000 per QALY. Our study may offer evidence to guide the assessment of therapeutic alternatives and pricing setting for advanced NSCLC.

## Data availability statement

The original contributions presented in the study are included in the article/Supplementary material, further inquiries can be directed to the corresponding authors.

## Author contributions

YJ: Conceptualization, Data curation, Formal analysis, Investigation, Methodology, Writing – original draft, Writing – review & editing. MZ: Conceptualization, Data curation, Formal analysis, Investigation, Methodology, Writing – original draft, Writing – review & editing. JX: Data curation, Supervision, Writing – review & editing. JL: Investigation, Methodology, Writing – review & editing. WT: Supervision, Writing – review & editing, Funding acquisition, Resources, Validation, Visualization. XZ: Investigation, Methodology, Supervision, Validation, Visualization, Writing – review & editing.
