# Supplementary material for: Cost-effectiveness analysis of atezolizumab in patients with non-small-cell lung cancer ineligible for treatment with a platinum-containing regimen: a United Kingdom health care perspective
Source: Front Public Health. 2023 Sep 29;11:1282374. doi: 10.3389/fpubh.2023.1282374 (PMC10570614; doi:10.3389/fpubh.2023.1282374)
Supplement: Supplementary file 1 [file Data_Sheet_1.DOCX]

**Supplement**

[Figure S1 Model Structure 2](#_Toc3158)

[Table S1 Baseline characteristics of patients in the IPSOS trial 3](#_Toc7459)

[Table S2 Subsequent anti-cancer therapies 4](#_Toc13010)

[Table S3 Model choice for all survival data 5](#_Toc15670)

[Table S4 Goodness-of-fit results 6](#_Toc4264)

[Figure S2 Visual validation 8](#_Toc32678)

[Table S5 Adverse events (AE, grade ≥ 3) occurrences, unit costs of AE management 10](#_Toc29471)

[Table S6 Health state utility values and disutilites for AE 11](#_Toc8714)

[Table S7 Disutility and duration of adverse event 12](#_Toc22836)

[Table S8 Drug administration costs 13](#_Toc2085)

[Table S9 Drug acquisition unit costs in the model 14](#_Toc23920)

[Table S10 Healthcare resource use and unit costs 15](#_Toc22762)

[Table S11 Cost of best supportive care 18](#_Toc24715)

[Table S12 Summary of variables applied in the economic model 20](#_Toc472)

[Figure S3 Summary results for scenario 6 21](#_Toc18473)

[Table S13 Breakdown results of costs 22](#_Toc15484)

[Table S14 Summary results for scenarios 1-5 23](#_Toc15484)

# Figure S1 Model Structure


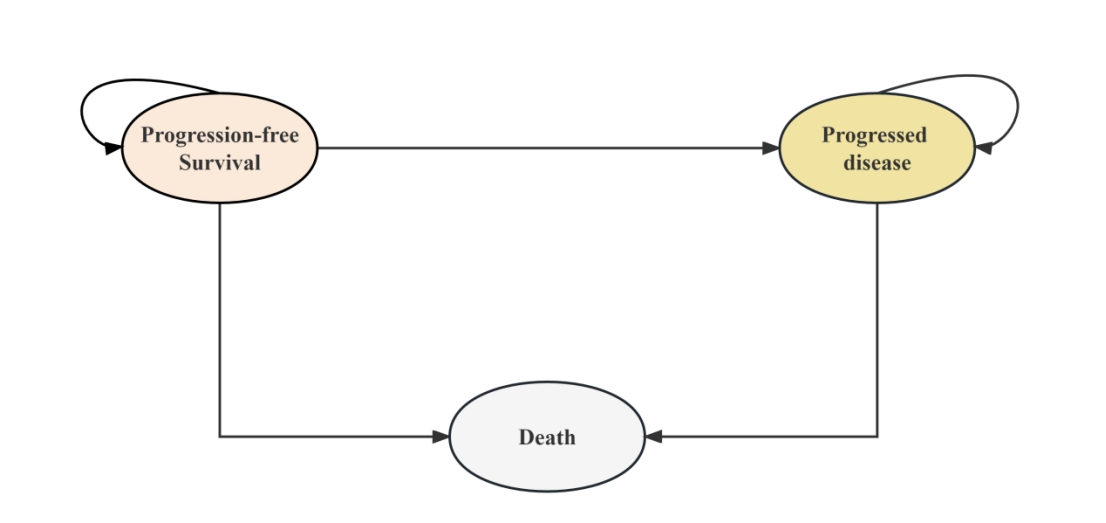


# Table S1 Baseline characteristics of patients in the IPSOS trial[1]

| Baseline characteristics for patients in the IPSOS trial | | |
| --- | --- | --- |
|  | Atezolizumab (N=320) | Chemotherapy (N=151) |
| Age, median (range), years | 75 (69–81) | 75 (68–80) |
| Male | 220 (73%) | 108 (72%) |
| Race |  |  |
| White | 203 (67%) | 95 (63%) |
| Asian | 75 (25%) | 38 (25%) |
| ECOG Performance Status Scale scored |  |  |
| 0-1 | 56 (19%) | 19 (13%) |
| 2 | 228 (75%) | 116 (77%) |
| 3 | 18 (6%) | 16 (11%) |
| Tobacco use history |  |  |
| Previous | 209 (69%) | 103 (68%) |
| Current | 58 (19%) | 28 (19%) |
| Never | 35 (12%) | 20 (13%) |
| Histology |  |  |
| Non-squamous | 173 (57%) | 87 (58%) |
| Squamous | 129 (43%) | 64 (42%) |
| PD-L1 expression level |  |  |
| < 1% | 151 (50%) | 61 (40%) |
| ≥ 1%-<50% | 77 (25%) | 53 (35%) |
| ≥50% | 50 (17%) | 25 (17%) |
| Stage |  |  |
| ⅢB | 41 (14%) | 21 (14%) |
| Ⅳ | 261 (86%) | 130 (86%) |
| Brain metastases |  |  |
| Yes | 27 (9%) | 13 (9%) |
| No | 273 (90%) | 137 (91%) |
| Liver metastases |  |  |
| Yes | 44 (15%) | 26 (17%) |
| No | 258 (85%) | 125 (83%) |
| Number of metastatic sites |  |  |
| Less than 3 | 124 (41%) | 73 (48%) |
| ≥3 | 141 (47%) | 59 (39%) |
| EGFR mutation status |  |  |
| No mutation | 269 (89%) | 131 (87%) |
| ALK mutation status |  |  |
| No mutation | 266 (88%) | 130 (86%) |

ECOG PS Eastern Cooperative Oncology Group performance status score; PD-L1 Programmed cell death-ligand 1; EGFR, epidermal growth factor receptor; ALK, anaplastic lymphoma kinase.

# Table S2 Subsequent anti-cancer therapies for patients in the atezolizumab and chemotherapy arms

| Group | Atezolizumab*  (n=302) | Chemotherapy* (n=151) | Source |
| --- | --- | --- | --- |
| Carboplatin, N (%) | 16 (5) | 7 (5) | [1] |
| Pemetrexed, N (%) | 15 (5) | 4 (3) |  |
| Gemcitabine, N (%) | 16 (5) | 2 (1) |  |
| Docetaxel, N (%) | 5 (2) | 3 (2) |  |
| Vinorelbine, N (%) | 7 (2) | 1 (1) |  |
| Paclitaxel, N (%) | 5 (2) | 2 (1) |  |
| Nivolumab, N (%) | 0 | 13 (9) |  |
| Atezolizumab, N (%) | 0 | 9 (6) |  |
| Pembrolizumab, N (%) | 0 | 5 (3) |  |

*For patients received no subsequent anti-cancer therapies (79% in the atezolizumab and 72% in the chemotherapy arm), best supportive care was used.

# Table S3 Model choice for all survival data

| Name | Parameters |
| --- | --- |
| OS of atezolizumab: Gompertz | Shape: -0.2343336/ Rate:-0.1015898 |
| OS of chemotherapy (base-case analysis): RP-odds (2 knots) | gamma0: 0.4057861/gamma1:1.7800612/ gamma2: -1.0921587/gamma3:2.4948455 gamma4:-2.2317045/gamma5:0.7018199 |
| PFS of atezolizumab (base-case analysis): RP-hazard (1 knot) | gamma0:7.5572635/gamma1:3.453652 gamma2:0.455467/gamma3:-0.5254197 gamma4:0.2313602 |
| PFS of chemotherapy: Log-normal | Meanlog: -1.08424654/ sdlog: -0.08455412 |
| OS of chemotherapy (scenario analysis): Log-normal | Meanlog:-0.353219521/sdlog:-0.002626766 |
| PFS of atezolizumab (scenario analysis): Log-normal | Meanlog:-1.0383328/sdlog:0.2102123 |

Note: OS: overall survival; PFS: progression-free survival; RP: Royston-Parmar models.

# Table S4 Goodness-of-fit results

| Model | LnL | Params | AIC | Model | LnL | Params | AIC |
| --- | --- | --- | --- | --- | --- | --- | --- |
|  | Overall Survival of Atezolizumab | | |  | Overall Survival of chemotherapy | | |
| gompertz | -122.08 | 2 | 248.15 | RP-odds-2 | -83.44 | 6 | 178.87 |
| FP2-2 | -121.46 | 3 | 248.92 | RP-normal-2 | -83.64 | 6 | 179.29 |
| RCS1 | -121.50 | 3 | 249.01 | RP-hazard-1 | -83.91 | 6 | 179.81 |
| FP2-1 | -121.56 | 3 | 249.12 | RP-normal-1 | -88.02 | 2 | 180.03 |
| weibull | -122.74 | 2 | 249.48 | lnorm | -88.02 | 2 | 180.03 |
| RP-hazard-2 | -122.04 | 3 | 250.08 | RP-odds-1 | -86.10 | 4 | 180.19 |
| gengamma | -122.06 | 3 | 250.13 | FP2-2 | -87.25 | 3 | 180.50 |
| RP-normal-1 | -122.12 | 3 | 250.23 | llogis | -88.28 | 2 | 180.56 |
| RCS2 | -121.49 | 4 | 250.98 | gengamma | -87.29 | 3 | 180.58 |
| RP-hazard-1 | -120.63 | 5 | 251.25 | FP1-2 | -88.52 | 2 | 181.04 |
| gamma | -123.71 | 2 | 251.41 | FP2-1 | -87.59 | 3 | 181.17 |
| RP-normal-2 | -120.86 | 5 | 251.73 | RP-hazard-2 | -86.62 | 4 | 181.23 |
| genf | -122.06 | 4 | 252.13 | genf | -87.29 | 4 | 182.58 |
| RP-odds-2 | -122.10 | 4 | 252.20 | FP1-1 | -89.57 | 2 | 183.14 |
| RP-odds-1 | -121.16 | 5 | 252.32 | gamma | -90.20 | 2 | 184.41 |
| FP1-1 | -124.34 | 2 | 252.69 | weibull | -91.27 | 2 | 186.54 |
| llogis | -124.44 | 2 | 252.89 | exp | -92.75 | 1 | 187.51 |
| lnorm | -125.12 | 2 | 254.24 | RCS1 | -90.87 | 3 | 187.73 |
| FP1-2 | -125.82 | 2 | 255.63 | RCS2 | -90.03 | 4 | 188.05 |
| exp | -127.29 | 1 | 256.58 | gompertz | -92.74 | 2 | 189.48 |
| Model | LnL | Params | AIC | Model | LnL | Params | AIC |
|  | Progression-free Survival of Atezolizumab | | |  | Progression-free Survival of chemotherapy | | |
| RP-hazard-1 | -111.73 | 3 | 229.46 | lnorm | -61.61 | 2 | 127.22 |
| RP-odds-2 | -111.74 | 4 | 231.47 | RP-normal-1 | -61.61 | 2 | 127.22 |
| lnorm | -110.46 | 6 | 232.93 | RP-hazard-1 | -59.70 | 4 | 127.40 |
| gengamma | -109.71 | 7 | 233.42 | RP-odds-1 | -60.50 | 4 | 129.01 |
| genf | -110.11 | 7 | 234.23 | RP-normal-2 | -61.74 | 3 | 129.49 |
| RP-normal-2 | -115.18 | 2 | 234.35 | gengamma | -61.81 | 3 | 129.63 |
| RP-hazard-2 | -110.30 | 7 | 234.61 | RP-hazard-2 | -61.93 | 3 | 129.85 |
| RP-odds-1 | -114.73 | 3 | 235.46 | RP-odds-2 | -60.32 | 5 | 130.65 |
| llogis | -117.32 | 2 | 238.64 | FP2-2 | -62.66 | 3 | 131.32 |
| RP-normal-1 | -116.95 | 3 | 239.91 | genf | -61.81 | 4 | 131.63 |
| FP2-2 | -118.12 | 3 | 242.24 | FP1-2 | -64.07 | 2 | 132.14 |
| gompertz | -117.26 | 4 | 242.53 | llogis | -64.24 | 2 | 132.47 |
| FP2-1 | -124.30 | 2 | 252.61 | FP2-1 | -64.41 | 3 | 134.81 |
| RCS1 | -128.92 | 2 | 261.84 | FP1-1 | -66.75 | 2 | 137.50 |
| RCS2 | -133.49 | 2 | 270.98 | RCS2 | -65.44 | 4 | 138.89 |
| weibull | -134.84 | 1 | 271.69 | gamma | -67.67 | 2 | 139.33 |
| gamma | -137.26 | 2 | 278.52 | weibull | -69.38 | 2 | 142.75 |
| FP1-1 | -111.73 | 3 | 229.46 | exp | -71.50 | 1 | 144.99 |
| exp | -111.74 | 4 | 231.47 | gompertz | -71.36 | 2 | 146.72 |
| FP1-2 | -110.46 | 6 | 232.93 | RCS1 | -71.26 | 3 | 148.52 |

Note: OS: overall survival; PFS: progression-free survival; LnL: log likelihood; Params: Parameters; AIC: Akaike information criterion; Exp: exponential; lnorm: log normal; llogis: log logistic; gengamma: generalized gamma; FP: fractional polynomial; RCS: restricted cubic spline models; RP: Royston-Parmar models.

# Figure S2 Visual validation

Overall survival for atezolizumab (Gompertz model was chosen)


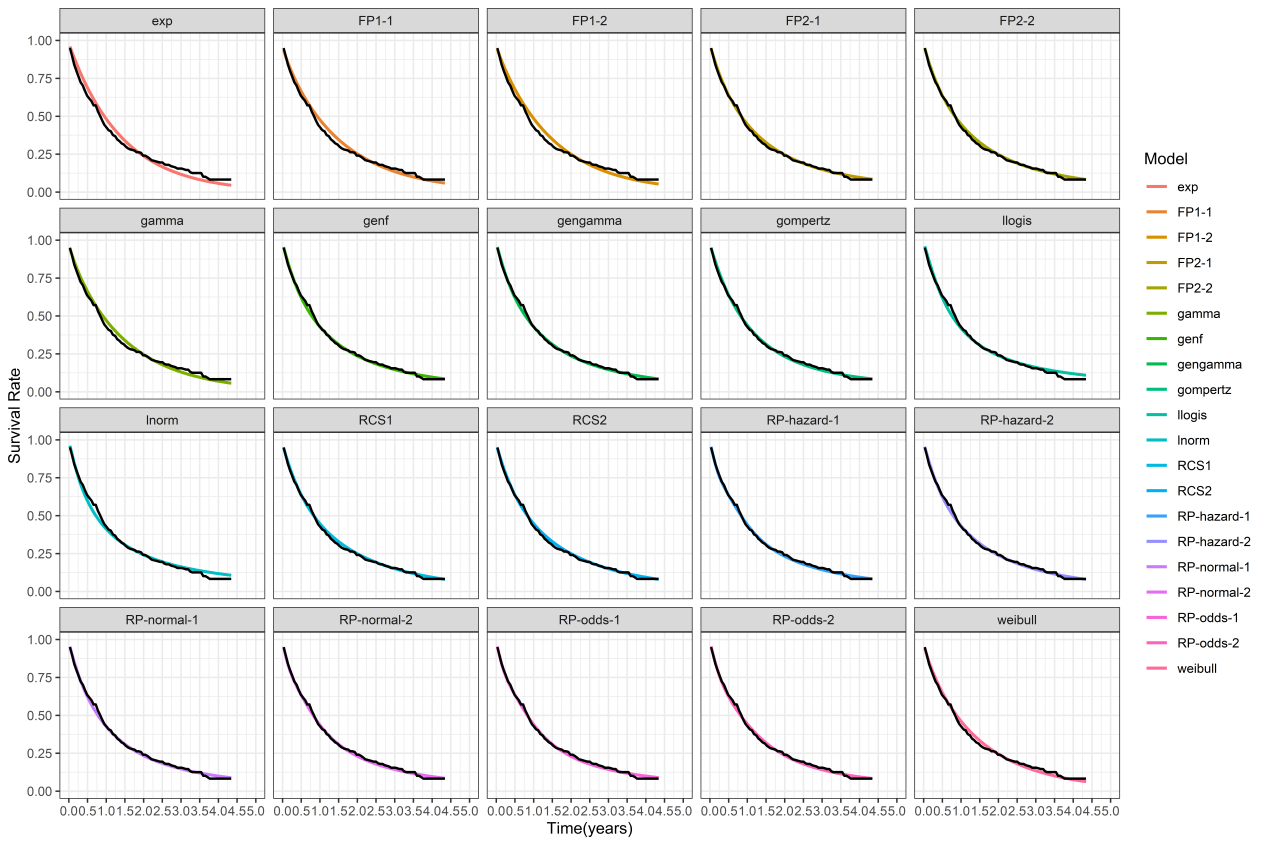


Overall survival for chemotherapy (RP-odds model was chosen for the base-case analysis, Lognormal model was chosen for scenario analysis)


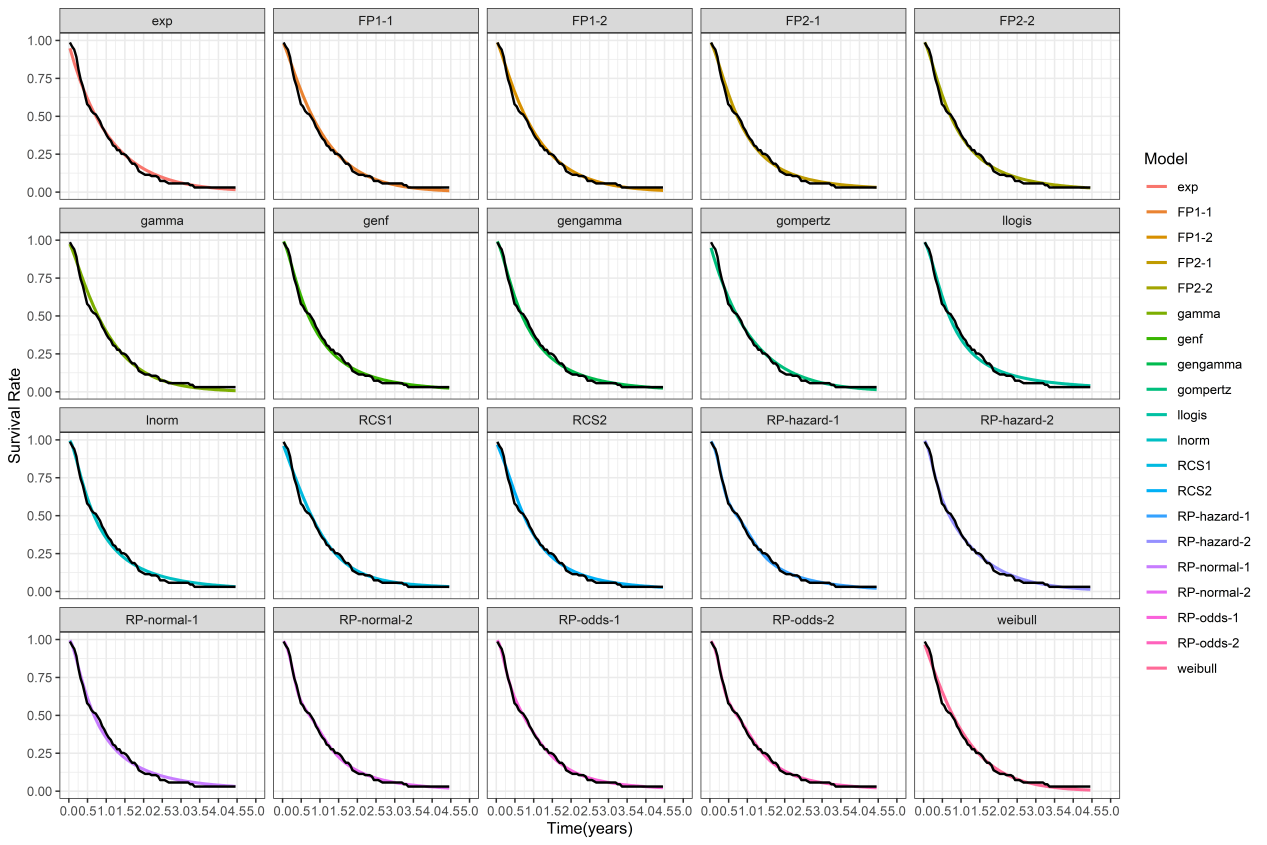


Progression-free survival for atezolizumab (RP-hazard model was chosen for the base-case analysis, Lognormal model was chosen for scenario analysis)


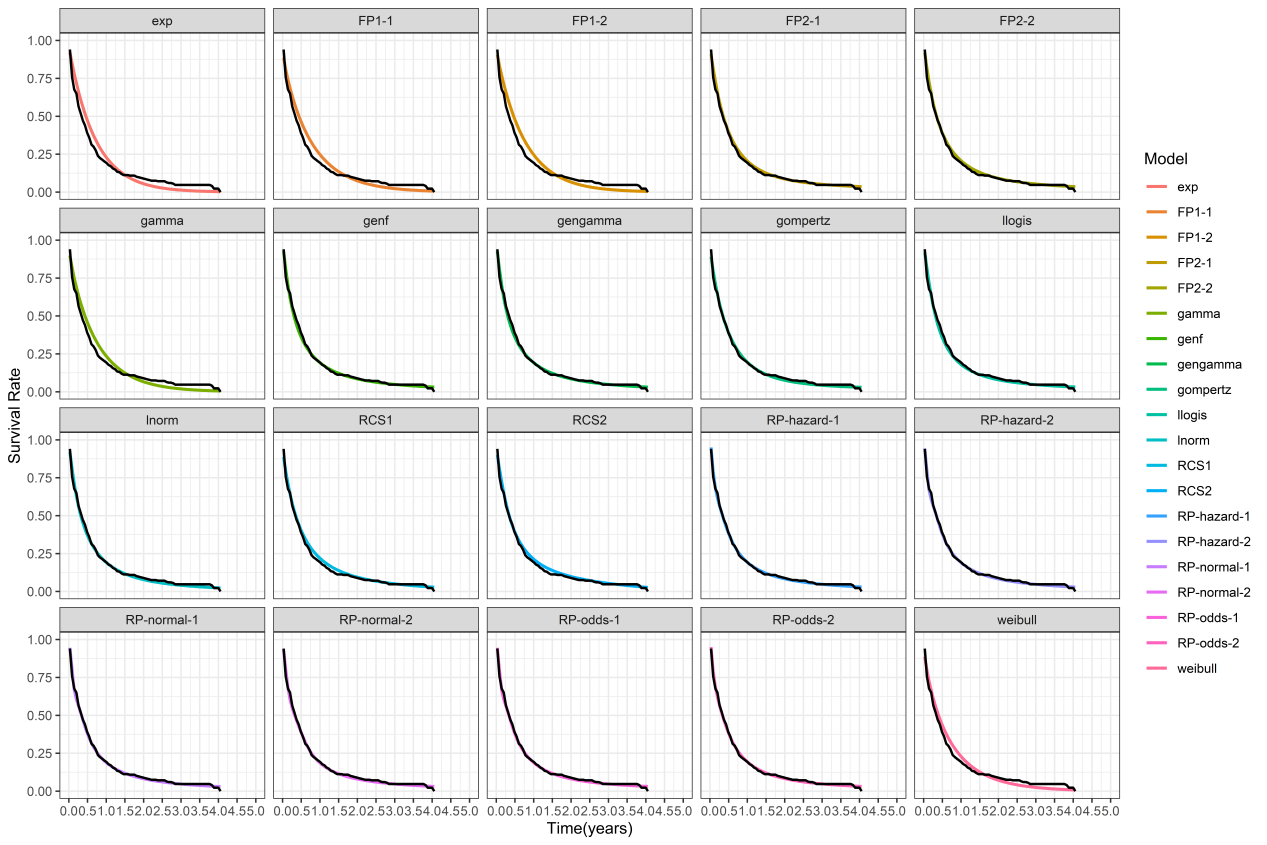


Progression-free survival for chemotherapy (Log-normal model was chosen)


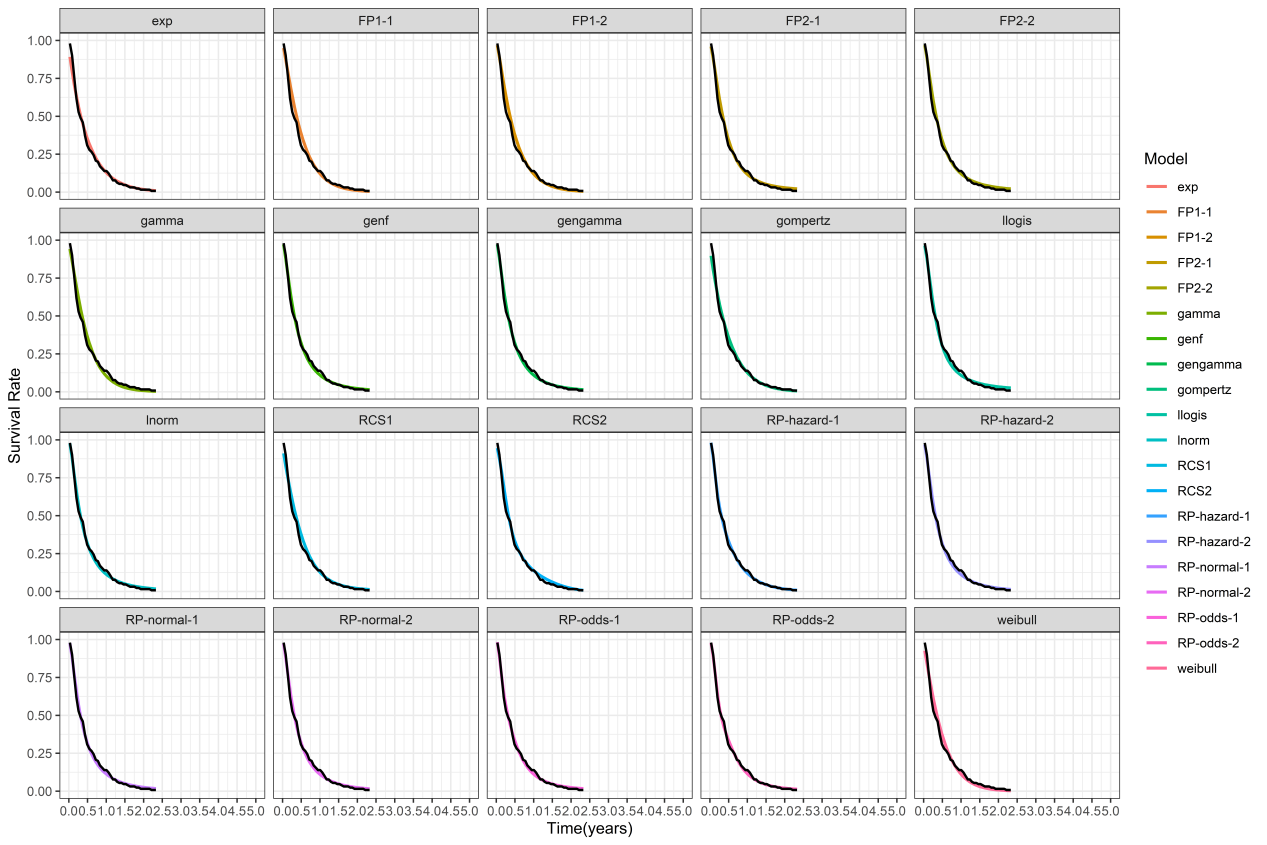


# Table S5 Adverse events (AE, grade ≥ 3) occurrences, unit costs of AE management. Rates were cited from the IPSOS trial

| Adverse events | Incidence (atezolizumab) | Incidence (chemotherapy) | Cost/£ | Source |
| --- | --- | --- | --- | --- |
| Dyspnoea | 0.05 | 0.04 | 684.17 | NHS Reference Cost 2022: Weighted average of Total HRGs  DZ19H (Other Respiratory Disorders) |
| Anaemia | 0.03 | 0.04 | 753.00 | NHS Reference Cost 2022: SA05J |
| Neutropenia | 0.01 | 0.1 | 660.81 | NICE TA705- inflated to 2022 using the PSSRU HCHS index |
| Leukopenia | 0 | 0.02 | 414.14 | NICE TA531 - inflated to 2022 using the PSSRU HCHS index |
| Nausea | 0.01 | 0.015 | 1770.00 | NHS Reference Cost 2022: PF28C; NICE TA876 |
| Vomiting | 0 | 0.01 | 1770.00 | NHS Reference Cost 2022: PF28C |
| Rash | 0.01 | 0 | 1115.00 | NHS Reference Cost 2022: PJ66A |
| Neutrophil count decreased | 0 | 0.08 | 277.28 | NICE TA909 |
| White blood cell count decreased | 0 | 0.04 | 493.87 | NICE TA705- inflated to 2022 using the PSSRU HCHS index |

Abbreviations: NHS, National Health Service; PSSRU, Personal social service research unit in the UK; NICE, National institute for Health and Care Excellence.

# Table S6 Health state utility values and disutilites for AE

| Parameter | Utility (SE) | Disutility | Source |
| --- | --- | --- | --- |
| Utility for gerenal people (age: 75, percent of male: 73%) | 0.76 | / | [2] |
| Treatment (PFS, base case analysis) | 0.71 (0.01) | 0.11 | [3] |
| No treatment (PFS) | 0.62 (0.03) | 0.17 | [4] |
| Treatment (PD, base case analysis) | 0.69(0.02) | 0.13 | [3] |
| No treatment (PD) | 0.62 (0.03) | 0.17 | [4] |
| Treatment (PFS, scenario analysis) | 0.76 (0.01) | 0.05 | [5] |
| Treatment (PD, scenario analysis) | 0.69 (0.02) | 0.11 | [5] |

# Table S7 Disutility and duration of adverse event

| AE | Disutility | Source | Duration (day) | Source |
| --- | --- | --- | --- | --- |
| Dyspnoea | 0.05 | NICE TA911 | 18.8 | NICE TA544 |
| Anaemia | 0.08973 | NICE TA876 | 3 | NICE TA544 |
| Neutropenia | 0.08973 | [6] | 158 | NICE TA544 |
| Leukopenia | 0.08973 | NICE TA876 | 15 | NICE TA812 |
| Nausea | 0.04802 | [6] | 10.5 | NICE TA544 |
| Vomiting | 0.04802 | [6] | 2 | NICE TA544 |
| Rash | 0.03 | [6] | 117.6 | NICE TA544 |
| Neutrophil count decreased | 0 | [7] | 0 | NICE TA911 |
| White blood cell count decreased | 0 | [7] | 4.5 | [8] |

Table S8 Drug administration costs

| Drug | Type of administration | Cost per administration (£) | Source |
| --- | --- | --- | --- |
| All iv therapies^*^ | Deliver simple parenteral chemotherapy at first attendance | 287 | NHS reference costs 2021-2022 SB12Z |

*In line with the NICE TA823 appraisal, zero administration cost is assumed

Abbreviations: IV: intravenous injection; NHS, National Health Service.

# Table S9 Drug acquisition unit costs in the model

| Drugs used in the model | Method and frequency of administration | Dose per vial/pack (large vial, mg) | Dose size | Cost per vial/pack (£) | | Source |
| --- | --- | --- | --- | --- | --- | --- |
|  |  |  |  | Mean | SD |  |
| Carboplatin | IV, Q3W | 600 | 150mg AUC | 21.32 | 18.06 | eMIT 2022 Code DHA162 |
| Pemetrexed | IV, Q3W | 500 | 500mg/m^2^ | 450 | NA | BNF (accessed 26 July 2023) |
| Gemcitabine | IV, Q3W | 2200 | 1,250 mg/m² twice | 45.9 | 4.5 | eMIT 2022 Code DHB249 |
| Docetaxel | IV, Q3W | 160 | 75 mg/m² | 15.67 | 2 | eMIT 2022 Code DHC046 |
| Vinorelbine | IV, Q3W | 50 | 30 mg/m² twice | 159.59 | 22.07 | eMIT 2022 Code DHA221 |
| Vinorelbine | Oral, Q3W | 80 | 60 mg/m² for first 3 cycles, 80 mg/m² then | 95.58 | 2.07 | eMIT 2022 Code DHA338 |
| Paclitaxel | IV, Q3W | 300 | 200 mg/m^2^ | 19.85 | 14.62 | eMIT 2022 Code DHA210 |
| Nivolumab | IV, Q3W | 240 | 3 mg/kg once every 2 weeks | 2633 | NA | BNF (accessed 26 July 2023) |
| Atezolizumab | IV, Q3W | 1200 | 1200mg | 3807.69 | NA | BNF (accessed 26 July 2023) |
| Pembrolizumab | IV, Q3W | 100 | 200mg | 2630 | NA | BNF (accessed 26 July 2023) |

Abbreviations: AUC: area under receiver operating characteristics curve; BNF, British National Formulary; eMIT: electronic market information tool; IV: intravenous injection; NA, not available; SD, standard deviation.

# Table S10 Healthcare resource use and unit costs (Healthcare resource costs for patients in the first-line and second-line states were £326 and £506 per cycle)

| Healthcare resource | 1L treatment-visits/hours per year | 2L treatment-visits/hours per year | Unit costs/£ | Cost per month for first-line | Cost per month for second-line | Unit cost reference |
| --- | --- | --- | --- | --- | --- | --- |
| Ct chest scan | 4 scans | 0 scans | 142 | 47 | 47 | NHS Reference Costs 2022, diagnostic lmaging, Outpatient, HRG code RD24Z |
| Chest radiography | 6.79 scans | 6.50 scans | 34 | 19 | 18 | NHS Reference Costs 2022: Code 370 outpatient medical oncology, per visit. |
| Electrocardiogram | 1.04 scans | 0.88 scans | 159 | 14 | 12 | NHS Reference Costs 2022, Complex ECG, HRG code EY51Z, service Code 370, per scan. |
| Outpatient visit | 9.61 visits* | 7.91 visits* | 235 | 188 | 155 | Band 8a, Cost per hour. PSSRU 2022 |
| Community nurse | 8.70 visits* | 8.70 visits* | 89 | 65 | 65 | Band 8b, Cost per hour. PSSRU 2022 |
| Clinical nurse specialist | 12 visits* | 12 visits* | 96 | 96 | 96 | Cost per hour. PSSRU 2022. |
| GP surgery | 12 visits | 0 visits | 41 | 41 | 0 | NHS Reference Costs 2022: Code 370 outpatient medical oncology, per visit. |
| GP home visit | 0 visits | 26.09 visits | 105 | 0 | 227 | PSSRU 2016: Cost per home visit including 11.4 minutes for consultations and 12 minutes for travel (NICE TA531, inflated using the PSSRU HCHS index 2022) |
| Therapist visit | 0 visits | 26.09 visits | 50 | 0 | 109 | PSSRU 2022; cost per hour for community occupational therapist (including training) |

*UK clinical oncologists assumed that a visit would be ~1 hour, therefore we assumed one hour per visit.

Abbreviations: NHS, National Health Service; PSSRU, Personal social service research unit in the UK; GP, general practitioner

# Table S11 Cost of best supportive care

| Category | Dose | Unit cost (£) | Per cycle cost (£) | Source |
| --- | --- | --- | --- | --- |
| Radiotherapy |  | 152.88 | 28.67 | NHS Reference Costs (2018/19); Total Outpatient Attendances, 800, Clinical Oncology (previously radiotherapy), inflated using the PSSRU HCHS index 2022 |
| Steroids | 0.5mg daily | 2.71 | 13.84 | [Drugs and pharmaceutical electronic market information tool (eMIT 2020; https://www.gov.uk/government/publications/drugs-and-pharmaceutical-electronic-market-information-emit)](nttps://www.gov.uk/government/publications/drugs-and-), inflated using the PSSRU HCHS index 2022 |
| NSAIDs(aspirin) | 75mg daily | 0.32 | 0.06 | [Drugs and pharmaceutical electronic market information tool (eMIT 2020; 75mg tablets, 100 pack, pack cost f0.30; nttps://www.gov.uk/government/publications/drugs-and- pharmaceutical-electronic-market-information-emit](nttps://www.gov.uk/government/publications/drugs-and-)), inflated using the PSSRU HCHS index 2022 |
| Morphine | 40-60mg daily | 28.48 | 10.68 | [Drugs and pharmaceutical electronic market information tool (eMIT) 2020; Morphine 50mg tablets / Packsize 56 cost f26.56; nttps://www.gov.uk/government/publications/drugs-and- pharmaceutical-electronic-market-information-emit](nttps://www.gov.uk/government/publications/drugs-and-)), inflated using the PSSRU HCHS index 2022 |
| Bisphosphonate (alendronic acid) | 10mg daily | 0.97 | 0.72 | [Drugs and pharmaceutical electronic market information tool (eMIT) 2020; Alendronic acid 10mg tablets/Packsize 28, pack cost E0.90; nttps://www.gov.uk/government/publications/drugs-and- pharmaceutical-electronic-market-information-emit](nttps://www.gov.uk/government/publications/drugs-and-)), inflated using the PSSRU HCHS index 2022 |
| Denosumab | 120mg every 4 weeks | 196.22 | 294.33 | BNF Accessed February 2020, inflated using the PSSRU HCHS index 2022 |
| Dietitian |  | 96.40 | 352.04 | NHS Reference Costs (2018/19); CHS, AHP, A03, Dietitian, inflated using the PSSRU HCHS index 2022 |

# Table S12 Summary of variables applied in the economic model

| Parameter | Mean | Lower value | Higher value | Distribution | Reference to section |
| --- | --- | --- | --- | --- | --- |
| Body Surface Area (m2) | 1.850 | 1.665 | 2.035 | gamma | NICE TA823 |
| Body weight (kg) | 74.070 | 66.66 | 81.48 | gamma | NICE TA823 |
| AUC | 6 | 5.4 | 6.6 | gamma | NICE TA823 |
| Withdraw rate per cycle (atezolizumab) | 0.023 | 0.018 | 0.028 | beta | IPSOS |
| Withdraw rate per cycle (chemotherapy) | 0.037 | 0.030 | 0.044 | beta | IPSOS |
| Discount for cost and utility | 0.035 | 0.015 | 0.050 | beta | NICE TA823 |
| Percent of Gemcitabine | 0.500 | 0.400 | 0.600 | beta | Assumed |
| Male percent | 0.73 |  |  | Fiexed | IPSOS |
| Age | 75.00 |  |  | Fiexed | IPSOS |
| Administration and follow-up cost | | | | | |
| Follow-up_cost_first-line | 326.00 | 260.80 | 391.20 | gamma | Table S6 |
| Follow-up_cost_second-line | 505.98 | 404.78 | 607.17 | gamma |  |
| Drug administration cost | 287.00 | 229.60 | 344.40 | gamma | Table S5 |
| Dyspnoea_Cost | 684.17 | 547.34 | 821.00 | gamma | Table S7 |
| Anaemia_Cost | 753.00 | 602.40 | 903.60 | gamma |  |
| Neutropenia_Cost | 660.81 | 528.65 | 792.98 | gamma |  |
| Leukopenia_Cost | 414.14 | 331.31 | 496.96 | gamma |  |
| Nausea_Cost | 1770 | 1416 | 2124 | gamma |  |
| Vomiting_Cost | 1770 | 1416.00 | 2124 | gamma |  |
| Rash_Cost | 1115 | 892.00 | 1338.00 | gamma |  |
| Neutrophil count decreased_Cost | 277.28 | 221.82 | 332.74 | gamma |  |
| White blood cell count decreased_Cost | 493.87 | 395.09 | 592.64 | gamma |  |
| End of life cost | 4772.7 | 4025.73 | 5610.919 | gamma | NICE TA823 |
| Best supportive care | 378.77 | 303.019 | 454.528 | gamma | Table S8 |
| Utility and disutility | | | | | |
| PFS_on treatment_utility | 0.65 | 0.63 | 0.67 | beta | Table S9 |
| PFS_off treatment_utility | 0.59 | 0.53 | 0.65 | beta |  |
| PD_on treatment_utility | 0.63 | 0.59 | 0.67 | beta |  |
| PD_off treatment_utility | 0.59 | 0.53 | 0.65 | beta |  |
| Dyspnoea_disutility_utility | 0.05 | 0.04 | 0.06 | beta | Table S10 |
| Anaemia_disutility_utility | 0.09 | 0.07 | 0.11 | beta |  |
| Neutropenia_disutility | 0.09 | 0.07 | 0.11 | beta |  |
| Leukopenia_disutility | 0.09 | 0.07 | 0.11 | beta |  |
| Nausea_disutility | 0.05 | 0.04 | 0.06 | beta |  |
| Vomiting_disutility | 0.05 | 0.04 | 0.06 | beta |  |
| Rash_disutility | 0.03 | 0.02 | 0.04 | beta |  |
| Duration of AE | | | | | |
| Dyspnoea_duration | 18.80 | 15.04 | 22.56 | gamma | Table S10 |
| Anaemia_duration | 3.00 | 2.40 | 3.60 | gamma |  |
| Neutropenia_duration | 158.00 | 126.40 | 189.60 | gamma |  |
| Leukopenia_duration | 15.00 | 12.00 | 18.00 | gamma |  |
| Nausea_duration | 10.50 | 8.40 | 12.60 | gamma |  |
| Vomiting_duration | 2.00 | 1.60 | 2.40 | gamma |  |
| Rash_duration | 117.60 | 94.08 | 141.12 | gamma |  |
| Rate of AE | | | | | |
| Dyspnoea (atezolizumab) | 0.05 | 0.04 | 0.06 | beta | Table S7 |
| Anaemia (atezolizumab) | 0.03 | 0.02 | 0.04 | beta |  |
| Neutropenia (atezolizumab) | 0.01 | 0.01 | 0.01 | beta |  |
| Nausea (atezolizumab) | 0.01 | 0.01 | 0.01 | beta |  |
| Rash (atezolizumab) | 0.01 | 0.01 | 0.01 | beta |  |
| Dyspnoea (chemotherapy) | 0.04 | 0.03 | 0.05 | beta |  |
| Anaemia (chemotherapy) | 0.04 | 0.03 | 0.05 | beta |  |
| Neutropenia (chemotherapy) | 0.10 | 0.08 | 0.12 | beta |  |
| Leukopenia (chemotherapy) | 0.02 | 0.02 | 0.02 | beta |  |
| Nausea (chemotherapy) | 0.02 | 0.01 | 0.02 | beta |  |
| Vomiting (chemotherapy) | 0.01 | 0.01 | 0.01 | beta |  |
| Neutrophil count decreased(chemotherapy) | 0.08 | 0.06 | 0.10 | beta |  |
| White blood cell count decreased (chemotherapy) | 0.04 | 0.03 | 0.05 | beta |  |
| Subsequent anti-cancer therapies | | | | | |
| Carboplatin (atezolizumab) | 0.05 | 0.04 | 0.06 | beta | Table S6 |
| Pemetrexed (atezolizumab) | 0.05 | 0.04 | 0.06 | beta |  |
| Gemcitabine (atezolizumab) | 0.05 | 0.04 | 0.06 | beta |  |
| Docetaxel (atezolizumab) | 0.02 | 0.01 | 0.02 | beta |  |
| Vinorelbine (atezolizumab) | 0.04 | 0.03 | 0.04 | beta |  |
| Paclitaxel (atezolizumab) | 0.02 | 0.01 | 0.02 | beta |  |
| Carboplatin (chemotherapy) | 0.05 | 0.04 | 0.06 | beta |  |
| Pemetrexed (chemotherapy) | 0.03 | 0.02 | 0.03 | beta |  |
| Gemcitabine (chemotherapy) | 0.01 | 0.01 | 0.02 | beta |  |
| Docetaxel (chemotherapy) | 0.02 | 0.02 | 0.02 | beta |  |
| Vinorelbine (chemotherapy) | 0.03 | 0.02 | 0.03 | beta |  |
| Paclitaxel (chemotherapy) | 0.01 | 0.00 | 0.08 | beta |  |
| Nivolumab (chemotherapy) | 0.09 | 0.07 | 0.10 | beta |  |
| Atezolizumab (chemotherapy) | 0.06 | 0.00 | 0.08 | beta |  |
| Pembrolizumab (chemotherapy) | 0.03 | 0.03 | 0.04 | beta |  |

# Figure S3 Summary results for scenario 6


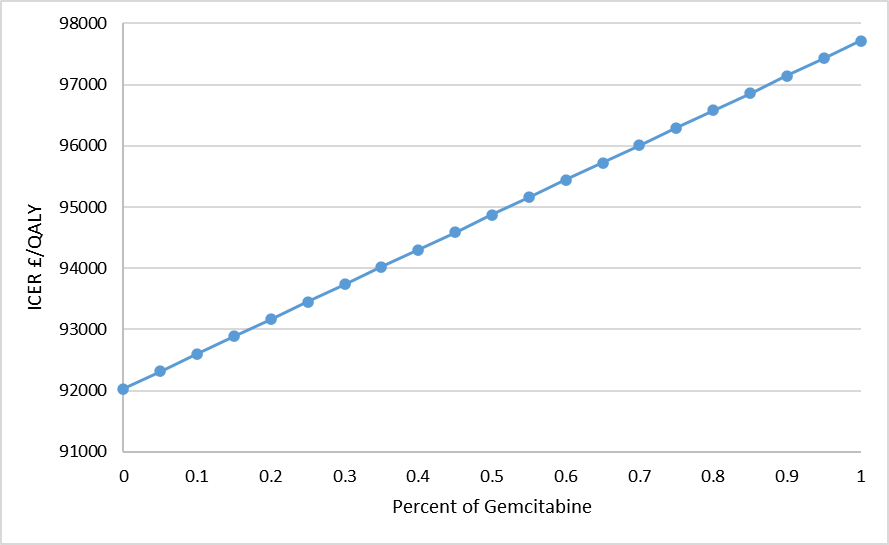


Abbreviations: ICER, incremental cost-effectiveness ratio.

# Table S13 Breakdown results of costs (￡)

| Cost breakdown | Drug | Follow-up | AE | Death | Total |
| --- | --- | --- | --- | --- | --- |
| atezolizumab | 41627.28 | 10661.30 | 92.2566439 | 4568.87 | 56949.71 |
| chemotherapy | 18197.53 | 7663.06 | 218.038054 | 4665.04 | 30743.67 |

# Table S14 Summary results for scenarios 1-5

| Scenario | Utility of chemotherapy | Cost of chemotherapy | Utility of atezolizumab | Cost of atezolizumab | ICER (£/QALY) |
| --- | --- | --- | --- | --- | --- |
| 1 | 0.60 | 31291 | 0.86 | 57536 | 99040 |
| 2 | 0.61 | 30733 | 0.90 | 56958 | 90974 |
| 3 | 0.58 | 30488 | 0.86 | 54970 | 88219 |
| 4 | 0.58 | 30490 | 0.86 | 56456 | 94001 |
| 5 | 0.59 | 29720 | 0.86 | 56908 | 97354 |

**References**

[1]. Lee, S.M., et al., First-line atezolizumab monotherapy versus single-agent chemotherapy in patients with non-small-cell lung cancer ineligible for treatment with a platinum-containing regimen (IPSOS): a phase 3, global, multicentre, open-label, randomised controlled study. Lancet, 2023.

[2]. Ara, R. and J.E. Brazier. Populating an economic model with health state utility values: moving toward better practice. Value Health, 2010. 13(5): p. 509-18.

[3]. Socinski, M.A., et al., Atezolizumab for First-Line Treatment of Metastatic Nonsquamous NSCLC. N Engl J Med, 2018. 378(24): p. 2288-2301.

[4]. van den Hout, W.B., et al., Cost-utility analysis of short- versus long-course palliative radiotherapy in patients with non-small-cell lung cancer. J Natl Cancer Inst, 2006. 98(24): p. 1786-94.

[5]. Jassem, J., et al., Updated Overall Survival Analysis from IMpower110: Atezolizumab Versus Platinum-Based Chemotherapy in Treatment-Naive Programmed Death-Ligand 1-Selected NSCLC. J Thorac Oncol, 2021. 16(11): p. 1872-1882.

[6]. Nafees, B., et al., Health state utilities for non-small cell lung cancer. Health Qual Life Outcomes, 2008. 6: p. 84.

[7]. Kuznik, A., et al., Cost-Effectiveness of Cemiplimab Versus Standard of Care in the United States for First-Line Treatment of Advanced Non-small Cell Lung Cancer with Programmed Death-Ligand 1 Expression ≥50. Value Health, 2022. 25(2): p. 203-214.

[8]. Shao, T., et al., Cost-effectiveness analysis of camrelizumab plus chemotherapy as first-line treatment for advanced squamous NSCLC in China. Front Public Health, 2022. 10: p. 912921.
